# Machine learning-driven discovery of high-performance MEMS disk resonator gyroscope structural topologies

**DOI:** 10.1038/s41378-024-00792-4

**Published:** 2024-10-30

**Authors:** Chen Chen, Jinqiu Zhou, Hongyi Wang, Youyou Fan, Xinyue Song, Jianbing Xie, Thomas Bäck, Hao Wang

**Affiliations:** 1 Xi’an Jiaotong University, Faculty of Electronic and Information Engineering, Xi’an, China; 2 Northwestern Polytechnical University, School of Mechanical Engineering, Xi’an, China; 3 Beijing Microelectronics Technology Institute, Beijing, China; 4 Leiden University, Leiden Institute of Advanced Computer Science, Leiden, Netherlands

**Keywords:** Electrical and electronic engineering, Electronic devices, Sensors

## Abstract

The design of the microelectromechanical system (MEMS) disc resonator gyroscope (DRG) structural topology is crucial for its physical properties and performance. However, creating novel high-performance MEMS DRGs has long been viewed as a formidable challenge owing to their enormous design space, the complexity of microscale physical effects, and time-consuming finite element analysis (FEA). Here, we introduce a new machine learning-driven approach to discover high-performance DRG topologies. We represent the DRG topology as pixelated binary matrices and formulate the design task as a path-planning problem. This path-planning problem is solved via deep reinforcement learning (DRL). In addition, we develop a convolutional neural network-based surrogate model to replace the expensive FEA to provide reward signals for DRL training. Benefiting from the computational efficiency of neural networks, our approach achieves a significant acceleration ratio of 4.03 × 10^5^ compared with FEA, reducing each DRL training run to only 426.5 s. Through 8000 training runs, we discovered 7120 novel structural topologies that achieve navigation-grade precision. Many of these surpass traditional designs in performance by several orders of magnitude, revealing innovative solutions previously unconceived by humans.

## Introduction

Microelectromechanical system (MEMS) gyroscopes, which use the Coriolis effect to detect the angular velocity or angle of an object’s rotation^1^, are core components of motion tracking and inertial navigation. MEMS gyroscopes offer substantial advantages over traditional mechanical gyroscopes, including miniaturization, cost efficiency, and lower power consumption. These benefits are critical for a wide range of applications, from emerging Internet of Things (IoT) technologies—such as mixed reality, sports motion tracking, and advanced medical devices—to high-end industrial uses in robotics, transportation, and space navigation.

**Fig. 1:**
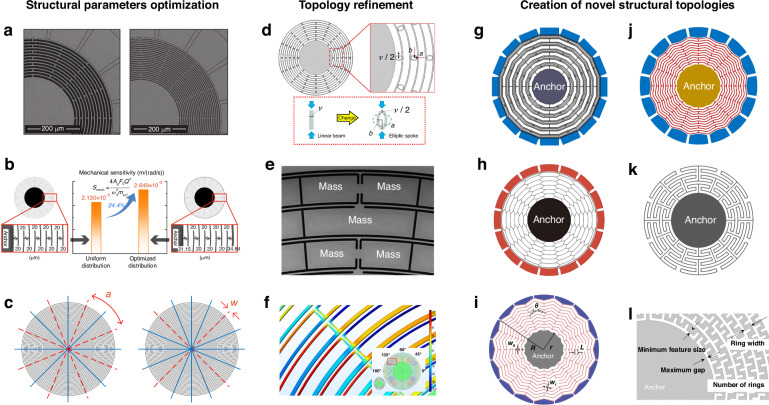
Design methods for improving MEMS DRG performance. **a** Enhancing the Q-factor by optimizing the ring width and spoke length^8^. **b** Improving the mechanical sensitivity via an optimized ring thickness distribution^9^. **c** Preventing frequency splitting by adjusting the spokes^3^. **d** Eliminating the stiffness anisotropy by incorporating elliptical spokes^40^. **e** Increasing the effective mass by resuspending lumped masses^10^. **f** Designing the thermal distribution of crank suspension beams^41^. **g** Cobweb-like DRG^11^. **h** Honeycomb-like DRG^12,13^. **i** Flower-like DRG^42^. **j** Gear-like DRG^43^. **k** Radially pleated ring DRG^44^. **l** Spring-like ring DRG^45^

Despite these advantages, the performance of MEMS gyroscopes is still inferior to that of traditional mechanical gyroscopes, thereby restricting their application. Therefore, improving their performance is highly valuable. Among various MEMS gyroscopes, the disc resonator gyroscope (DRG) is recognized as a promising candidate for high-performance MEMS gyroscopes^2–13^. As shown in Fig. 1, the MEMS DRG, characterized by its continuum structure with multiple concentric rings and radial symmetry, which is interconnected by alternating spokes and mounted on an anchor, demonstrates high sensitivity, excellent vibration resistance, minimal anchor loss, degenerate modes, and low-temperature sensitivity.

**Fig. 2:**
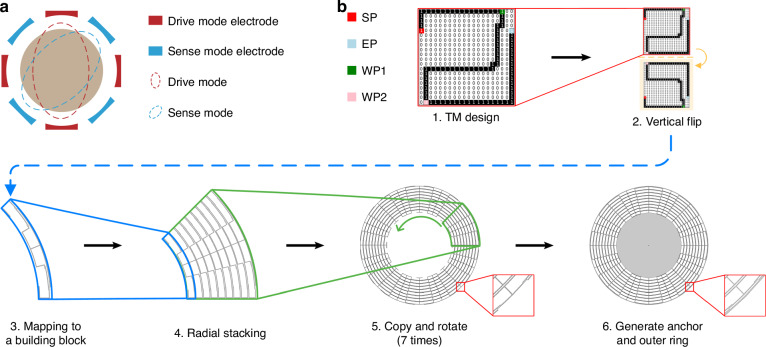
MEMS DRG configuration. **a** MEMS DRG operating conditions. The gyroscope operates in second-order degenerate mode, and the electrodes of the DRG can be placed peripherally or both peripherally and internally. **b** First, a continuous trajectory is designed in a trajectory matrix (TM). This trajectory originates from a starting point (SP) and concludes at an ending point (EP), ensuring that it passes through two waypoints (WP1 and WP2) before going to the EP. Second, this matrix undergoes a mirror flip operation and is concatenated with the original matrix. Third, the path in the rectangle-shaped TM can be homeomorphically mapped to the annular sector design block. Fourth, this design block undergoes radial scaling and stacking to form a 45° multilayer structure. Fifth, this multiring structure is copied circularly seven times to form a 360° complete multiring structure. Finally, an additional ring is added externally to create the complete DRG topology

The geometric design of MEMS DRGs can profoundly impact their physical properties and performance. Performance improvement can be achieved through three main strategies: (1) Structural parameter optimization, which involves parameterizing the design with several variables and identifying the optimal parameters, as illustrated in Fig. 1a, b; (2) topology refinement, which starts with a multiring topology (Fig. 1a) that is fine-tuned to meet specific objectives, as illustrated in Fig. 1d, e; and (3) the creation of novel structural topologies, which aims to optimize the material layout from scratch, as shown in Fig. 1g–l. Among these strategies, the creation of novel structural topologies is particularly notable for its potential to substantially enhance performance^5,11–14^. Nevertheless, innovating new topologies for MEMS DRGs presents considerable challenges. This involves not only the inherent complexity of the structural topology, which itself is a challenging NP-complete 0–1 integer programming problem^15^, but also a wide range of complicated microscale physical effects^1,5^. Additionally, evaluating the performance of MEMS DRG designs accurately is challenging; for example, accurate modeling of a MEMS DRG is very difficult^16,17^, and high-fidelity finite element analysis (FEA) simulations can be computationally intractable^18^. Consequently, these challenges have significantly hampered progress in MEMS DRG topology innovation over the last two decades.

As a potential solution to the topology design problem, data-driven machine-learning methods have been proposed for some MEMS devices, such as resonators, pressure sensors, and accelerometers. These approaches formulate a topology design problem either as a learning task^19–21^ or an optimization task^9,22–25^. Despite their innovative approaches, they often suffer from limited design flexibility and constrained geometries, severely limiting MEMS device performance. Moreover, the unavoidable reliance on expensive FEA for evaluating design performance has limited the number of explorations, often leading to locally optimal solutions. In addition to these common challenges in MEMS device topology design, some unique issues of MEMS DRGs, including strict geometric constraints, operating mode identification, and multistep FEA for performance evaluation, result in approaches that cannot be directly applied to MEMS DRGs.

In this work, we propose a novel machine learning-driven workflow for discovering high-performance MEMS DRG structural topologies. To facilitate machine learning-based design for the DRG topology, we introduce a novel representation of the DRG topology. In this representation space, we formulate the DRG topology design task as a path-planning problem that can be addressed effectively via deep reinforcement learning (DRL). Moreover, we develop a surrogate model based on convolutional neural networks to replace time-consuming FEA, thereby significantly accelerating the design evaluation process. This model enables each DRL training run time to be merely 426.5 s. Through DRL 8000 training runs, we successfully identified 7120 novel MEMS DRG topologies that achieve navigation-grade precision. The vast majority of discovered topologies outperform traditional designs, with some topologies achieving improvements in performance by orders of magnitude, many of which present innovative solutions previously unconceived by humans. We believe that this methodology can be applied to other MEMS devices, such as accelerometers, pressure sensors, microfluidic devices, and microphones.

## Results

### Design space of MEMS DRGs

In this work, we consider the second-order degenerate mode among all vibration modes of the DRG, as depicted in Fig. 2a: the mode shape, representing the vibration of the DRG, resembles two ellipsoids under drive and sense modes, which differ by 45 degrees in orientation. This mode offers a higher angular gain, lower mode frequency, and greater Coriolis mass, which are adopted in the vast majority of DRGs^5^. Additionally, the drive/sense electrodes of the DRG can be placed peripherally or both peripherally and internally with respect to the DRG (commonly adopted by Boeing^26^ and ADI^27^).

To enable the ML-driven design of DRGs, we need to develop a proper representation of the layout of DRG materials in space. We propose a building block representation in Fig. 2b: (1) As the drive and sense modes differ by 45°, we propose designing the layout in a 45° building block (step 3 in Fig. 2b). (2) Since both modes are symmetric, we need to ensure that the shape within the building block should also respect symmetry. Therefore, generating a design with half of the building block (namely, a 22.5° annular sector) is sufficient. (3) We create the layout of the 22.5° annular sector by first generating a continuous trajectory in a matrix (of size 20 × 20, denoted the trajectory matrix (TM) design). This TM is mirrored and then merged with itself and is subsequently continuously deformed into a 45° building block (step 3). Details of the mapping operation can be found in the Methods section. The TM design is a binary matrix where a value of ‘1’ indicates the presence of materials and vice versa. (4) To complete the 45° multilayer annular sector, we need to stack only single-layer building blocks along the radial axis multiple times (step 4). (5) We copy the 45° multilayer shape seven times to form a 360° multiring topology (step 5). (6) This topology is mounted on the internal anchors and enclosed by an external ring, which together form the boundary constraint to create the complete MEMS DRG topology. In this study, we keep the structural parameters of MEMS DRGs constant, including the diameter of the anchor, the overall outer diameter of the topology, the height of each building block, the number of layers, and the thickness. For more details, please see Supplementary Note S1. Notably, it is necessary to consider the following geometrical constraints for the TM design:

1. The path must remain confined within the boundaries of the TM.

2. The path should originate from a starting point (SP) and conclude at an ending point (EP), ensuring that it passes through two waypoints (WP1 and WP2) before going to the EP.

3. The SP randomly lies on the left boundary of the TM, whereas the EP is on the right boundary, maintaining identical vertical coordinates to ensure that the single 45° annular sectors are interconnected with each other when they are stacked radially.

4. The WP1 is located randomly on the top boundary, whereas the WP2 is randomly selected at the top boundary, ensuring that the annular sector is interconnected with when it is copied circularly.

5. Movement from one point is limited to approaching the next point either horizontally or vertically, thereby preventing the creation of an excessively irregular path.

Despite these constraints, the expressive power of the proposed design space is still strong, containing 7.2 × 10^27^ possible structural topologies (for the calculation, see Supplementary Note S2).

### Design of the MEMS DRG topology as a reinforcement learning problem

**Fig. 3:**
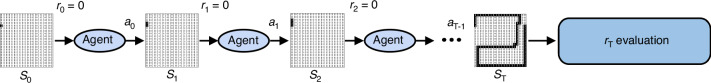
Overview of our method and training regimen. In each training iteration, the reinforcement learning (RL) agent moves a step at a time, eventually constructing a DRG topology (actions, states, and rewards are denoted *a*_*i*_, *s*_*i*_, and *r*_*i*_, respectively). The intermediate rewards are zero. The reward at the end of each trajectory is calculated as a linear combination of the evaluations from surrogate models

Considering the inherent complexity of the path-planning task within an enormous design space, deriving effective heuristic rules or design paradigms through domain knowledge becomes exceedingly challenging. Consequently, we propose generating the trajectory from a Markov decision process (MDP) and leveraging reinforcement learning (RL) to address it. RL involves training an agent to make decisions by interacting with an environment to maximize cumulative rewards over time. In our application, the RL agent starts from a random initialization of the four critical endpoints (SP, WP1, WP2, and EP) in the trajectory matrix. At each action step, the agent evaluates the current state and decides how to act next to ensure the trajectory’s validity and optimality. Our MDP consists of three key elements:

1. States encode information about the current path, the current coordinate of an agent, and the coordinates of four endpoints.

2. In each state, similar to the constraints in the design space, the agent chooses to either move horizontally or vertically to approach the next point.

3. As shown in Fig. 3, the rewards are 0 for all actions except the last action, which is typical in RL^28,29^. The final reward is the weighted sum of four performance metrics, as described below:

In this work, we consider four key performance metrics of a candidate topology in degenerate mode. (1) The quality factor (*Q*) measures the energy dissipation of the DRG; a higher value indicates less energy loss and thus higher resonator efficiency. (2) Mechanical sensitivity (*S*_*mech*_) influences the signal-to-noise ratio of the system, aiding in enhancing the accuracy and reliability of the gyroscope output. (3) Mechanical noise (*ARW*_*mech*_) indicates the DRG’s mechanical resolution, reflecting its ability to detect minute rotational changes. (4) The figure of merit (*FoM*) relates to the gyroscope’s bias stability, with a lower *FoM* being advantageous for reducing bias stability. Therefore, the final reward is defined as follows:

1 $$R=\alpha \cdot Q+\beta /FoM+\gamma \cdot {S}_{mech}+\epsilon /AR{W}_{mech}$$

where *α, β, γ*, and *ε* are four constants that serve as weighting factors to indicate the significance of each performance metric.

We train a policy (an RL agent) modeled by a neural network that learns to take actions that maximize the cumulative reward through repeated episodes (a sequence of states, actions, and rewards). Given the discrete nature of the action space, we utilize the SOTA RL method to learn the trajectory. Considering the discrete action space, we choose dueling double deep Q-network (D3QN)^30^ to address this task, primarily because of its enhanced stability and efficiency.

### Performance evaluation accelerated by a convolutional neural network

**Fig. 4:**
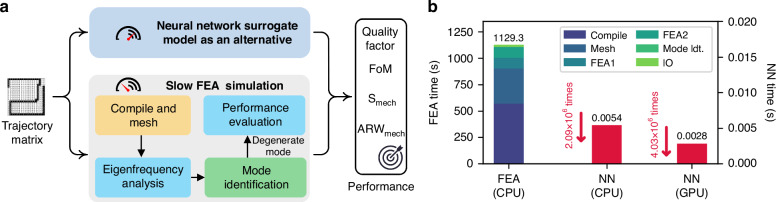
Comparison between the FEA and surrogate models. **a** Workflow of the performance evaluation of the DRG topology. The traditional finite element analysis approach involves several steps and is very time-consuming for high-fidelity simulations. We propose an alternative—a neural network-based surrogate model—to replace the slow FEA process. **b** Benchmarking the computation time of the FEA and the surrogate model approaches: for FEA, the simulation is conducted on an Intel Xeon Platinum 8163 CPU. ‘Compile’ represents the time consumed by converting input matrices into the internal variables of the FEA simulator (569.4 s), ‘Mesh’ represents FEA mesh division (332.5 s), ‘FEA1’ represents eigenfrequency analysis (101.6 s), ‘FEA2’ represents performance simulation (103.5 s), ‘Mode Idt.’ represents mode identification (0.031 s), and ‘IO’ represents the operation by which the FEA1 results are written to a SSD (22.3 s). For the surrogate model, it takes 5.4 ms on the same CPU used to test the FEA approach; it takes 2.8 ms on the NVIDIA RTX4090 GPU

The performance of each candidate topology (represented by the corresponding trajectory matrices) can be precisely computed via finite element analysis (FEA)^17,31,32^. To conduct this FEA, as shown in the gray box in Fig. 4a, candidates should take the following steps: compilation of the project, mesh division, eigenfrequency analysis, identification of the degenerate mode frequency, and multiphysics FEA using the mode frequency corresponding to the degenerate mode. Note that the degenerate mode frequency is typically identified by visually checking the mode shape of each eigenfrequency of the DRG topology. Owing to the need to automate this process, we construct a convolutional neural network for this task. The training dataset for this neural network (of size 2000) is sampled uniformly at random (by taking random actions at each step of the agent) and labeled by human experts. We then split this dataset into training and validation sets at a ratio of 8:2. The trained neural network achieved an accuracy rate of 99.77% on the validation set. We include the details of its architecture, training procedure, and data augmentation in the Methods section. This neural network can identify degenerate modes in merely 0.031 s on our CPU. We compared our method with two baselines, the window function method^33^ and Hu invariant moments^34^, finding accuracies of 1.32% and 3.02%, respectively. This poor performance is attributed to these methods being designed for specific topologies, resulting in limited generalizability across varying topologies.

Despite the high accuracy of FEA, the computational cost is very expensive. Given that DRL typically requires many evaluations of candidate DRGs to converge, directly using FEA to compute the performance metrics is intractable in our task. Alternatively, we propose the construction of highly accurate, cost-effective surrogate models for replacing FEA. The surrogate model takes the trajectory matrices as inputs and outputs the performance metrics, as shown in Fig. 4a. To generate the training data, we also sample 42,000 trajectory matrices uniformly at random and compute the performance metrics for each with the FEA approach. We then split the training data into training and validation sets at a ratio of 8:2. The details regarding the network’s architecture, training procedure, and data augmentation are provided in the Methods section. Instead of directly training surrogate models, we trained four individual surrogate models that target the quality factor, mode frequency, angular gain coefficient (*A*_*g*_), and effective mass (*m*_*eff*_). The *FoM*, *S*_*mech*_, and A*RW*_*mech*_ are calculated via Eqs. (2) to (6). Ultimately, on the validation set, the relative errors for *Q*, *FoM*, *S*_*mech*_, and A*RW*_*mech*_ are 2.42%, 3.42%, 3.42%, and 2.04%, respectively, with coefficients of determination (*R*^2^ score) of 0.99, 0.98, 0.99, and 0.98, respectively. Importantly, the surrogate model substantially reduces the computation time of the performance evaluation. Figure 4b compares the neural network’s forward pass runtime and FEA runtime. Compared with FEA, the surrogate models achieve an acceleration ratio of 2.09 × 10^6^ when executed on our CPU and 4.03 × 10^6^ when executed on our GPU.

### Structural topology discovery with deep reinforcement learning

**Fig. 5:**
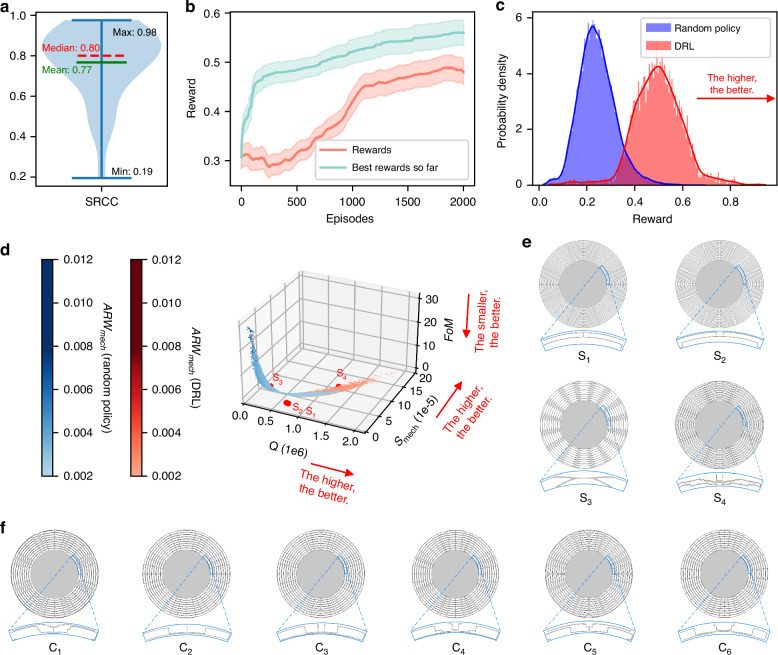
Optimization result of deep reinforcement learning. **a** SRCC between the predicted and actual rewards. **b** Convergence curves of RL. The curves are smoothed uniformly for clarity. The red curve represents the smoothed rewards, whereas the blue curve represents the smoothed optimal rewards obtained thus far. The shaded area in the graph represents the range of variation within one standard deviation. **c** Comparison of the ground truth rewards discovered via DRL and the random policy. **d** Distribution of performance for topologies discovered via DRL and the random policy (we removed data when the FoM value exceeded 30 to facilitate observation). **e** Traditional structural topologies in the proposed design space: S1 (multiring)^26^, S2 (crank-supported beam)^41^, and S3 (honeycomb)^12^ were obtained by manually planning agent paths. S4 (Gear-like)^43^ was discovered in the training dataset. Below each topology is the corresponding 45° single-ring topology. **f** Representative novel structural topologies from the top-20 rewards discovered via DRL. Below each topology is the corresponding 45° single-ring topology

In this study, the efficiency of agent training is enhanced by leveraging cost-effective data from surrogate models. However, there might be theoretical concerns about the distributional shift problem in model-based RL^35,36^, as the surrogate model is trained on randomly generated trajectories. When substituting FEA with surrogate models in the training of RL agents, we believe that it is necessary to validate the accuracy of the surrogate models via a small experiment. For the trajectories generated by the RL agent, we check whether the ranking of the final reward predicted by the surrogate model is consistent with that of the FEA approach, which is considered the ground truth. Since FEA is relatively time-consuming, we only compute the ground truth reward once every 100 episodes, on which we calculate the Spearman’s rank correlation coefficient (SRCC) between the surrogate model’s prediction and the corresponding reward from FEA. The distribution of the SRCC is shown in the violin plot in Fig. 5a; a median value of 0.8 strongly supports the use of the surrogate model in training. Additionally, we demonstrate the empirical convergence of training the RL agent in Fig. 5b, where the standard deviation is estimated from 30 runs.

The initial state of our RL approach is determined by the position of four key points (see Fig. 2b) in the trajectory matrix; we can enumerate all possible configurations (=8000 combinations) of the initial states when training the RL agent. In our training procedure, for each possible initial state, we train an RL agent with 2000 episodes/iterations and save the pair of the initial state and the trained agent. Among the 8000 training runs, there are 7264 feasible topologies that have the second-order degenerate mode, accounting for up to 90.80% of all the runs. Figure 5c compares the ground truth reward distributions between those produced by DRL and those produced by the random policy. The topologies designed by DRL generally outperform those generated randomly, demonstrating that DRL can converge effectively to high-performance topology regions under the guidance of surrogate models. Histograms of other performance metrics are presented in Supplementary Note S3. Regarding the FoM values, it is estimated that achieving navigation-grade zero bias stability requires an FoM ≤ 3.5°/s^37^ (as detailed in Supplementary Note S4). Among the randomly generated topologies, out of 36 361 feasible solutions, 17 647 topologies (48.53%) achieve navigation-grade accuracy, whereas 7 120 DRL-designed topologies (98.02%) achieve navigation-grade accuracy. Figure 5d illustrates the performance metric data distribution, where blue points denote randomly generated topologies and red points denote DRL-designed topologies. This demonstrates that even randomly generated structural topologies can achieve impressive performance, and the DRL-designed topologies are generally superior to those generated randomly, thereby confirming the efficiency of the proposed methodology. In Fig. 5e, we present four classical MEMS DRG topologies, which indicates that the proposed design space can cover classical structural topologies well.

Most topologies designed via DRL exhibit good performance. For demonstration, we select 6 representative topologies from the top-20 rewards, as shown in Fig. 5f. The performance metrics of the topologies in Fig. 5e, f are listed in Table 1. DRL-designed topologies outperform traditional topologies, especially in terms of the quality factor and FoM, where there is an order-of-magnitude improvement. The DRL-designed topologies are distinctly different from manually created topologies, offering greater flexibility and irregularity, with a higher degree of freedom. Remarkably, each exploration takes only 426.5 s, in contrast to the time required for human experts who, inspired by creative insight, often spend weeks or even months designing a new high-performance topology. More representative topologies can be found in Supplementary Note S5.

**Table 1:** Comparison of the mechanical performance metrics in Fig. 5e, f)

Topology	Q	*FoM* [°/s]	*S*_*mech*_ [m/(rad/s)]	ARW_mech_ [°/$\sqrt{h}$]	*f* [Hz]	*m*_*eff*_ [kg]	Reward
S_1_	8.68 × 10^5^	1.37	4.18 × 10^−5^	0.0042	5360.9	2.91 × 10^–7^	0.371
S_2_	7.70 × 10^5^	1.97	2.90 × 10^–5^	0.0027	6911.2	2.98 × 10^–7^	0.322
S_3_	4.07 × 10^5^	6.09	9.40 × 10^–6^	0.0031	10272.2	3.48 × 10^–7^	0.186
S_4_	1.40 × 10^6^	0.63	9.03 × 10^–5^	0.0027	3958.9	2.96 × 10^–7^	0.630
C_1_	2.15 × 10^6^	0.29	1.98 × 10^–4^	0.0025	2809.3	3.17 × 10^–7^	1.145
C_2_	2.00 × 10^6^	0.30	1.89 × 10^–4^	0.0025	2804.1	3.25 × 10^–7^	1.092
C_3_	1.83 × 10^6^	0.36	1.58 × 10^–4^	0.0025	3106.2	3.11 × 10^–7^	0.952
C_4_	1.99 × 10^6^	0.32	1.81 × 10^–4^	0.0025	2833.0	3.27 × 10^–7^	1.059
C_5_	1.80 × 10^6^	0.40	1.44 × 10^–4^	0.0024	3358.4	3.02 × 10^–7^	0.895
C_6_	1.83 × 10^6^	0.39	1.48 × 10^–4^	0.0024	3195.9	3.47 × 10^–7^	0.860

S_*n*_ represents classical structural topologies in the proposed design space. C_*n*_ represents new structural topologies discovered via DRL

**Fig. 6:**
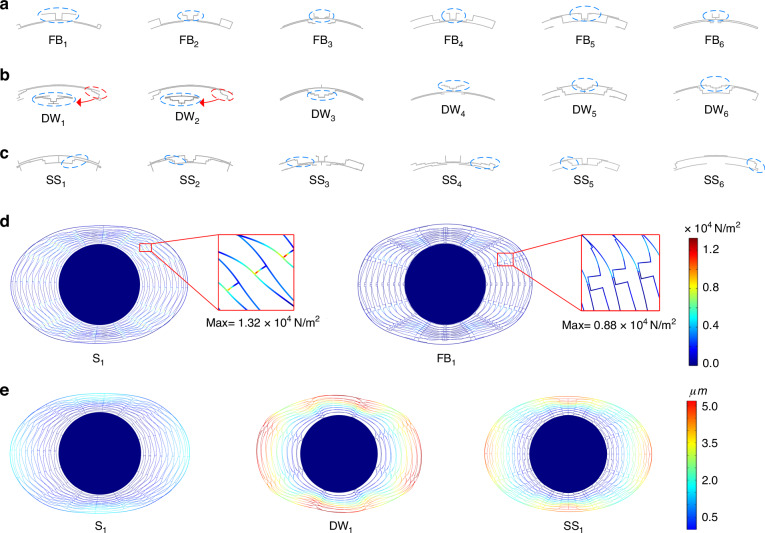
Examples of innovative design paradigms. **a** Examples of a flexible beam structure, surrounded with blue dashed lines. **b** Examples of a double-wing structure, surrounded with blue dashed lines. The double-wing structure can be formed by directly mirroring and flipping a 22.5° figure (as shown in the four images on the right). It can also be achieved by copying and rotating a 45° figure and then joining it with the original figure (as shown in the two images on the left). **c** Examples of a stepwise staircase structure, surrounded with blue dashed lines. **d** Analysis of the stress improvement in the flexible beam structure. **e** Deformation of the double-wing support structure and the stepwise staircase structure

By analyzing DRL-designed high-performance topologies, we observed several innovative structural elements (or design paradigms) that significantly enhance the mechanical performance. These discoveries, including flexible beam structures (as shown in Fig. 6a), double-wing structures (Fig. 6b), and stepwise staircase structures (Fig. 6c), can assist engineers in designing advanced topologies. As demonstrated in Fig. 6d, taking flexible beam structure FB_1_ as an example, compared with direct-connected spokes in multiring topologies, flexible beam structure FB1 can achieve a 33.2% reduction in maximum stress. Moreover, the stress concentration point also shifts from the rigid connection to the flexible connection. Compared with the multiring topology, when the double-wing structure DW_1_ and stepwise staircase SS_1_ are taken as examples, a greater degree of deformation is observed (as shown in Fig. 6e). This indicates a significant reduction in overall stiffness, which is beneficial for achieving a lower FoM value. The performance metrics of these topologies can be found in Supplementary Note S6.

## Discussion

To overcome the challenges of structural topology innovation in MEMS DRGs, this paper describes an innovative machine learning-driven workflow to discover high-performance MEMS DRG structural topologies. This workflow comprises three key components: a MEMS DRG design space, surrogate models to substitute for the expensive FEA, and DRL for structural topology discovery. Among these, DRL is the core of this workflow. We formulate the topology design task as a path planning problem, thereby using DRL to discover high-performance candidates within the design space. To expedite DRL training, this study employs convolutional neural network-based surrogate models to replace the time-consuming FEA to provide cheap reward signals for DRL training. Remarkably, after 8000 training runs, 7120 novel structural topologies that achieve navigation-grade performance have been identified, most surpassing traditional designs, with some even extending beyond the thinking patterns of human experts. With the acceleration capabilities of neural network surrogate models, each exploration takes an average of only 426.5 s—a stark contrast to that of the traditional manual design process, which is painstakingly slow, takes weeks or months, and depends heavily on designers’ expertise and creativity. Additionally, the study explores various design paradigms favored by the algorithm, offering valuable insights for engineers in creating advanced structural topologies.

MEMS DRGs are notably more complex than other MEMS devices because of their strict geometric constraints, multiple degrees of freedom, and the need for comprehensive multistep FEA to evaluate performance on the basis of specific operating modes. Therefore, the workflow we propose in this paper holds potential for broader application in the MEMS field, fostering advancements in device design and simulation methodologies. Moreover, an essential future direction for this research is the thorough integration of the entire design-to-manufacturing process. This integration will not only cover electrical sensitivity settings and fabrication parameters but also include manufacturability evaluation, ensuring that high-performance prototypes retain their performance when transitioning from simulation models to actual manufactured devices.

## Methods

### DRG mapping process

**Fig. 7:**
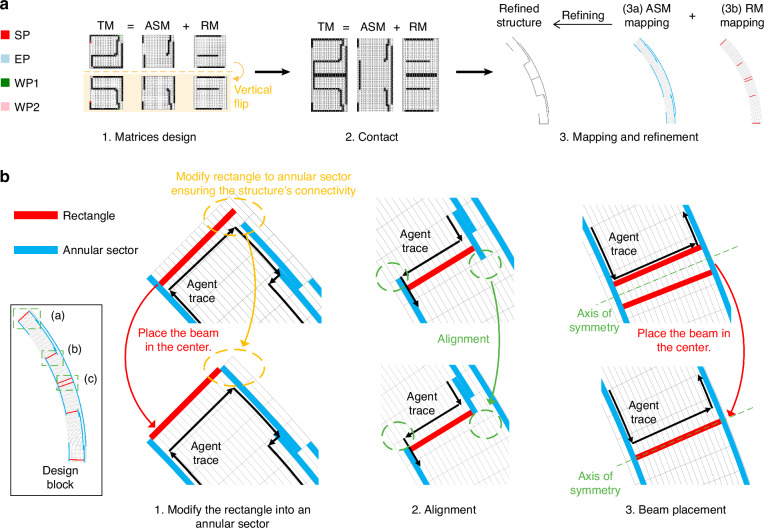
Mapping process for the design block. **a** Mapping process from a pixelated design space to a design block. First, three 20 × 20 matrices representing the layout are designed by generating a continuous trajectory. Second, all the matrices undergo mirror flipping and are concatenated with the original matrix to form a 40 × 20 matrix. Third, each ‘1’ element in the ASM is mapped to a small annular sector, and each ‘1’ in the RM is mapped to a small rectangle, culminating in a 45° annular sector structure. **b** Refinement operation. **b1** Modification of the rectangle into an annular sector to avoid disconnected topology. **b2** Structural alignment for manufacturing considerations. **b3** Prevention of redundant mapping of the central beam

We employ an agent to navigate a binary matrix, creating a path representing a structural topology. As shown in Fig. 7a, this process produces three fundamental matrices to represent a DRG topology: the trajectory matrix (TM), the annular sector matrix (ASM), and the rectangle matrix (RM), whose relationships are defined by the equation TM = ASM + RM. When the agent moves vertically (radially), it marks the corresponding position in the ASM with a ‘1’. Similarly, when it moves horizontally (tangentially), it marks the corresponding position in the RM with a ‘1’. As shown in Fig. 7a3, each ‘1’ element in the ASM is mapped into a small annular sector unit with an angle of 1.125° and a height of 10 µm. Each ‘1’ element in the RM is mapped into a small rectangular unit with both a length and height of 10 µm. The reason for not using a single TM to characterize the entire topology but rather using the ASM to characterize annular sectors and the RM to characterize rectangles separately is to reduce energy loss and thereby improve the DRG’s quality factor.

To ensure the continuum topology and to consider the feasibility of manufacturing, a refinement operation is conducted:

(1) When the agent changes its direction, for example, from vertical to horizontal (from radial to tangential), the corresponding shape switches from a rectangle to an annulus, as illustrated by the orange circle in Fig. 7b1. Consequently, the structure becomes discontinuous. Therefore, when the path changes direction, we modify the rectangular unit at the previous position into an annular sector unit to maintain the connectivity of the topology, as shown by the orange circle in Fig. 7b1.

(2) As the radius increases, the size of the annular sector unit increases, whereas the size of the rectangular unit remains constant. This discrepancy leads to a noticeable misalignment in the junction of the annular sector and the rectangle. To increase the feasibility of manufacturing, the topology is refined to ensure alignment between two shapes, as shown by the green circle in Fig. 7b2.

(3) The central beam is replicated during the mapping process. As a result, instead of mirroring the beam, we place it at the central mirror axis, as shown in Fig. 7b1, b3.

### System architecture summary

**Fig. 8:**
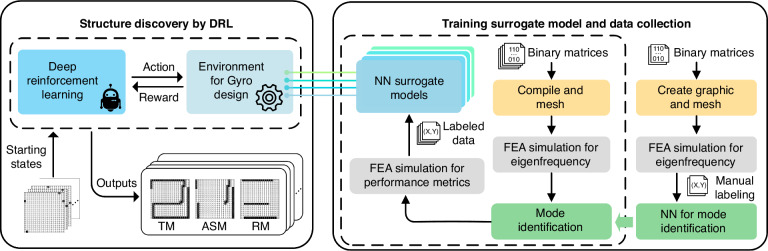
System architecture. The right side of the figure illustrates the training process for the surrogate models and a mode identification neural network. The left side of the figure illustrates the process of discovering a structural topology via reinforcement learning

We summarize the overall workflow from an implementation perspective in Fig. 8. The workflow consists of two major components: (1) a DRL agent that discovers novel structural topologies and (2) a deep neural network that serves as a surrogate model for performance evaluation acceleration.

The left side of Fig. 8 shows the discovery of a DRG topology via DRL. Before each exploration, the initial state (i.e., the positions of the SP, the EP, the WP1, and the WP2) is predefined. After each episode, the surrogate models evaluate the current design, and the environment calculates the reward. Finally, the candidates are the optimal topology from 2,000 explorations, characterized by the TM, ASM, and RM. The candidates from the DRL are further verified through FEA to evaluate the ground truth performance.

The right side shows the training process for the surrogate models and mode identification neural network. Each sample comprises binary matrices as input features (structural topologies) and performance metrics as labels, forming a training dataset for the surrogate model neural network. As shown by the dashed frame, to train this surrogate model, we employ a random policy to generate 42,000 input features within the design space. To obtain the labels of each input feature, each sample undergoes five steps, compile, mesh division, intrinsic frequency analysis, mode identification, and finite element multiphysics simulation, at the frequencies corresponding to degenerate modes. To automate this collection of training data, we not only employ scripts to invoke the FEA simulator but also implement a CNN to address mode identification as a binary classification problem, as shown by the green box in Fig. 8. To train the mode identification neural network, we randomly collect 2000 samples, simulate the first N-order intrinsic frequencies for each topology, and manually annotate the required degenerate modes. A CNN for mode identification is then trained on these manually annotated datasets.

### Mode identification neural network

We propose a convolutional neural network (CNN) technique to formulate the mode identification task as a binary classification problem. For each sample, we simulate the first N-order eigenfrequencies for each topology. The modes exhibiting the desired second-order degenerate mode are treated as positive samples, whereas other modes are considered negative. Data augmentation techniques are also employed to ensure a balanced class, which is detailed in the subsequent section.

For the mode identification neural network architecture, as shown in Supplementary Note S7, the input to the neural network consists of displacement field images and mode shape images for a given mode. The output is a Boolean value indicating whether the mode is the required degenerate mode. The total number of trainable parameters of this mode identification neural network is 123,610. Displacement field images and mode shape images are input into separate convolutional layers for feature extraction, after which the features are flattened, concatenated, and fed into the fully connected layers.

There are four convolutional layers, and the input channels of the convolutional layer are 1, 6, 6, and 16. The shape of the fully connected layer is 240-80-1. The network is trained via the Adam optimizer, with a fixed learning rate of 0.001. BCELoss is set as the loss function. The training process spans over 50 epochs with a batch size of 64. We include the details of its performance, including the convergence curve, in Supplementary Note S8. We use an 8:2 split ratio for data division for the training and validation sets.

### Neural network surrogate model

We collect 42,000 samples for neural network training, of which 36,361 (87.4%) exhibit the required second-order degenerate mode. For the samples lacking the required mode, we set their performance labels to 0.

For the surrogate model neural network architecture, as shown in Supplementary Note S7, the inputs are three matrices, the TM, ASM, and RM, which together represent the DRG topology, and the output is one performance metric. After the matrices pass through several residual blocks, the two-dimensional features are flattened into one-dimensional vectors. A fully connected layer subsequently transforms the features into performance. The detailed training convergence information can be found in Supplementary Note S9.

Our neural network is built with three residual blocks, whose input channels are 8, 8, and 16. Following each convolutional layer, the network includes a batch normalization layer as a regularization method. The total number of trainable parameters of this surrogate model is 12,449. The network is trained via the Adam optimizer, with a variable learning rate strategy starting at 0.001. The mean squared error (MSE) is set as the loss function. The training process spans over 300 epochs with a batch size of 64. For data division, we use an 8:2 split ratio for the training and validation sets.

### Data augmentation

#### Data augmentation for the mode identification neural network

For the training dataset of the mode identification neural network, we randomly generate 2000 topologies and then conduct FEA to obtain the mode shapes up to the *N*th order. The required degenerate mode is annotated as positive samples, whereas the remaining samples are labeled negative. In any given topology, only two of the N modes are positive, leaving the remaining (N-2) negative, which causes an imbalanced data problem. To address this issue, we rotate the positive samples multiple times within 45°. Such operations increase the number of positive samples, balancing the number of positive and negative samples in our dataset. This approach not only solves the issue of imbalance but also enriches the diversity of the training samples, which is crucial for the effective training of neural networks in mode identification tasks.

#### Data augmentation for the neural network surrogate model

Training neural networks benefit from a larger number of samples. In our case, owing to the inherent symmetry in the topologies, the topology mapped from the mirror-flipped matrices is equivalent to the original topology being rotated by 22.5°, with their performance remaining unchanged. This allows us to effectively double the number of training samples through mirror flipping.

### Dataset collection

Our platform is based on MATLAB and Python, with the commercial software COMSOL 5.6 used for FEA. Neural networks and reinforcement learning algorithms are developed ana analyzed via the PyTorch 2.0.1 platform. Through the API interface between MATLAB and COMSOL, we automatically create a geometric topology, perform mesh division, and run simulations. We use COMSOL’s “Free Triangular” function for mesh division. The material of the DRG is <111> polycrystalline silicon with a density of 2.3 × 10^3 ^kg/m^3^. The stiffness matrix references are obtained from the literature^38,39^.

Our experiments are carried out using an Intel Xeon Platinum 8163 CPU, 64 GB of memory, and an NVIDIA RTX 4090 GPU. During the training dataset collection phase, the GPU resources are not used. The GPU is used for only training neural network surrogate models and reinforcement learning tasks. The neural networks we used are of moderate size, allowing both their training and the optimization of reinforcement learning algorithms to be effectively performed on cheaper GPUs, such as the RTX 3060 laptop GPU.

On the basis of FEA, we collect 50,000 data points, which include 42,000 data points for training the surrogate models and 8000 data points for validating the performance of the topologies designed by DRL, consuming a total of 15,684.8 CPU hours. The DRL explorations, involving 8000 explorations, take 947.8 GPU hours. To improve efficiency, we employ several multiples for parallel processing acceleration.

### Datasets for the mode identification neural network

The neural network used for mode identification receives two input images: displacement images and mode shape images. These images are constructed using the coordinates (*X*_*n*_*, Y*_*n*_) and the displacement (Δ*X*_*n*_, Δ*Y*_*n*_) of each element, where *n* represents the *n*th element. These data are derived from FEA.

Displacement images are generated directly on the basis of the displacement data (Δ*X*_*n*_, Δ*Y*_*n*_). With respect to mode shape images, the displacement values (Δ*X*_*n*_, Δ*Y*_*n*_) are relatively small compared with the overall value of the coordinates (Δ*X*_*n*_, Δ*Y*_*n*_). To clearly visualize the modal shapes, these displacement values must be amplified, which is achieved with a factor of α = 40 × 10^4^. Consequently, the coordinates for the finite elements are recalculated as (*X*_*n*_ + *α*Δ*X*_*n*_*, Y*_*n*_ + *α*Δ*Y*_*n*_).

### Datasets for surrogate models

In the randomly collected training data, because the *ARW*_*mech*_, *S*_*mech*_, and *FoM* exhibit a long-tail distribution (see Supplementary Note S3), instead of directly training a surrogate model, we first fit some intermediate variables and then calculate them through physical formulas to obtain higher accuracy. After completing the eigenfrequency simulation, we employ Eqs. (2) and (3) to calculate the effective mass and angular gain coefficient.

2 $${m}_{eff}={\iiint_V}\rho ({\phi }_{x1}^{2}+{\phi }_{y1}^{2}+{\phi }_{z1}^{2}){{d}}V={\iiint_V}\rho ({\phi }_{x2}^{2}+{\phi }_{y2}^{2}+{\phi }_{z2}^{2}){{d}}V$$

3 $${A}_{g}=\frac{{\iiint_V}\rho ({\phi }_{x1}{\phi }_{y2}-{\phi }_{x2}{\phi }_{y1})dV}{2{m}_{eff}}$$

Given that the primary damping source for DRGs is thermoelastic damping (accounting for 64−80% of total damping^8^), we employ multiphysics simulations combining solid mechanics and thermodynamics to evaluate performance.

4 $$AR{W}_{mech}=\frac{1}{2{A}_{g}{x}_{0}}\sqrt{\frac{{k}_{B}{T}_{0}}{2\pi {f}_{0}{m}_{eff}Q}}\left(\frac{180}{\pi }\times 60\right)$$

5 $${S}_{mech}=\frac{4{A}_{g}Q{x}_{0}}{2\pi {f}_{0}}$$

6 $$FoM=\frac{1}{2{A}_{g}\tau }=\frac{\pi f}{2{A}_{g}Q}=\frac{\pi f}{2{A}_{g}Q}\times \frac{180}{\pi }$$

In these equations, *K*_*B*_ represents the Boltzmann constant, *T*_*0*_ denotes the temperature, and *x*_*0*_ is the normalized displacement.

### Deep reinforcement learning

For the Q-value neural network architecture, as shown in Supplementary Note S7, the neural network receives eight matrices representing state information processed by convolutional layers. Unlike natural images, which contain abundant redundant information, the topology represented by binary matrices is very concise when pooling is performed in convolutional layers. To prevent the loss of vital information, we employ convolutional layers with a stride of 1 for dimensionality reduction. Following the convolutional layers, the fully connected layer has two streams to estimate the state value and the advantages of each action separately. The output of the *Q*-network is the *Q*-value of each action. An additional term with an expected value of zero is added to the Q value, as shown in Eq. (7), to address the identifiability issue. The total number of trainable parameters of this Q-network is 17827.

7 $$Q({s}_{t},{a}_{t};{{\boldsymbol{\theta }}}_{t})=V({a}_{t};{{\boldsymbol{\theta }}}_{t})+A({s}_{t},{a}_{t};{{\boldsymbol{\theta }}}_{t})-\mathop{mean}\limits_{a}(A({s}_{t},{a}_{t};{{\boldsymbol{\theta }}}_{t}))$$

where ***θ***_*t*_ are the parameters (weights) of the Q-network at iteration *t*. In the *i*th iteration, D3QN updates via the loss function as follows:

8 $$L(\theta )={{\mathbb{E}}}_{({s}_{t},{a}_{t},{r}_{t},{s}_{t+1}) \sim U(D)}[{(({r}_{t}+\gamma Q({s}_{t+1},\mathop{\text{arg}\max }\limits_{a\in A}Q({s}_{t+1},a;{{\boldsymbol{\theta }}}_{t});{{\boldsymbol{\theta }}}_{t}^{-}))-Q({s}_{t},{a}_{t};{{\boldsymbol{\theta }}}_{t}))}^{2}]$$

in which *γ* is the discount factor determining the agent’s horizon, (*s*_*t*_*, a*_*t*_*, r*_*t*_*, s*_*t+1*_) ∼ *U(D)* is a mini-batch of experiences sampled from the replay buffer, and ${\boldsymbol{\theta}}_{t}^{-}$ represents the target network parameters used to compute the target at iteration t.

The number of steps of an episode is determined by the positions of the four endpoints. The size of the replay buffer is 1 × 10^6^. To further improve the stability of training, reward clipping is applied, and its lower and upper bounds are set to 0 and 3.5, respectively. During training, the Adam optimizer with a fixed learning rate of 0.001 and a batch size of 512 is employed. To balance exploration and exploitation, an epsilon-greedy strategy is utilized for exploration, with epsilon (exploration probability) set to 0.1.

## Supplementary information

Supplemental Video

Supplementary information

## Data Availability

The code for the mode identification neural network structure, the surrogate model structure, and the training and environment setup for reinforcement learning can be found in our GitHub repository (https://github.com/EchoChenC/MEMS_DRG).
