# Supplementary material for: Machine learning-driven discovery of high-performance MEMS disk resonator gyroscope structural topologies
Source: Microsyst Nanoeng. 2024 Oct 30;10:161. doi: 10.1038/s41378-024-00792-4 (PMC11522492; doi:10.1038/s41378-024-00792-4)
Supplement: Supplementary file 2 — Supplementary information [file 41378_2024_792_MOESM2_ESM.docx]

# Supplementary Information

## Supplementary Note S1: Parameters of the MEMS DRG

The size of the TM matrix is chosen to balance the grid resolution and decision space. Too low a resolution results in coarse topologies, whereas too high a resolution leads to dimensionality problems, complicating optimization. Additionally, we consider the limitations of manufacturing tolerances; an excessively high grid resolution might exceed the capabilities of the manufacturing process. In our study, the minimum size for the annular sector is set at 43.175 μm, which is derived from ((1.125°/360°)×2×2200 µm), and the minimum side length for a rectangle is 10 μm. These dimensions are chosen on the basis of the typical Bosch deep silicon etching process, which supports an etching aspect ratio of approximately 25:1, ensuring high production yield and robust process margins.

**Table R1.** Parameters of the DRG

| Parameters | Value |
| --- | --- |
| TM size | 20×20 |
| Anchor radius | 2200 µm |
| Angle of each annular sector unit | 1.125° |
| Height of each annular sector unit | 10 µm |
| Width of each rectangle unit | 10 µm |
| Height of each rectangle unit | 10 µm |
| Outer ring thickness | 20 µm |
| Single ring height | 20×8 µm |
| Number of layers | 9 |
| MEMS DRG radius | 3660 µm |
| MEMS DRG thickness | 100 µm |

## Supplementary Note S2: Calculation of the Design Space Size

In the context of our design, calculating the size of the design space is a traditional combinatorial problem. Assuming that the starting point and the endpoint are (*i*, 0) and (*i*, 19) and that the WP1 is (0, *j*) and the MWP2 is (19, *k*), the number of steps required to move from the starting point to the WP1 is *i + j*, with *j* steps in the vertical direction. This results in a total of $\left(\begin{matrix} i+j \\ j \end{matrix}\right)$ possibilities. Similarly, there are $\left(\begin{matrix} 19+|j-k| \\ |j-k| \end{matrix}\right)$ possibilities to move from WP1 to WP2 and $\left(\begin{matrix} 38-i-j \\ 19-k \end{matrix}\right)$ possibilities to move from WP2 to the endpoint. The calculations yield a design space size of 7.2×10^27^ possibilities.

$\sum_{i=0}^{19} \sum_{j=0}^{19} \sum_{k=0}^{19} \left(\begin{matrix} i+j \\ i \end{matrix}\right)\left(\begin{matrix} 19+|j-k| \\ |j-k| \end{matrix}\right)\left(\begin{matrix} 38-i-j \\ 19-k \end{matrix}\right)$ (R1)

## Supplementary Note S3: Comparison of Performance Metrics Generated by Reinforcement Learning and Random Policy


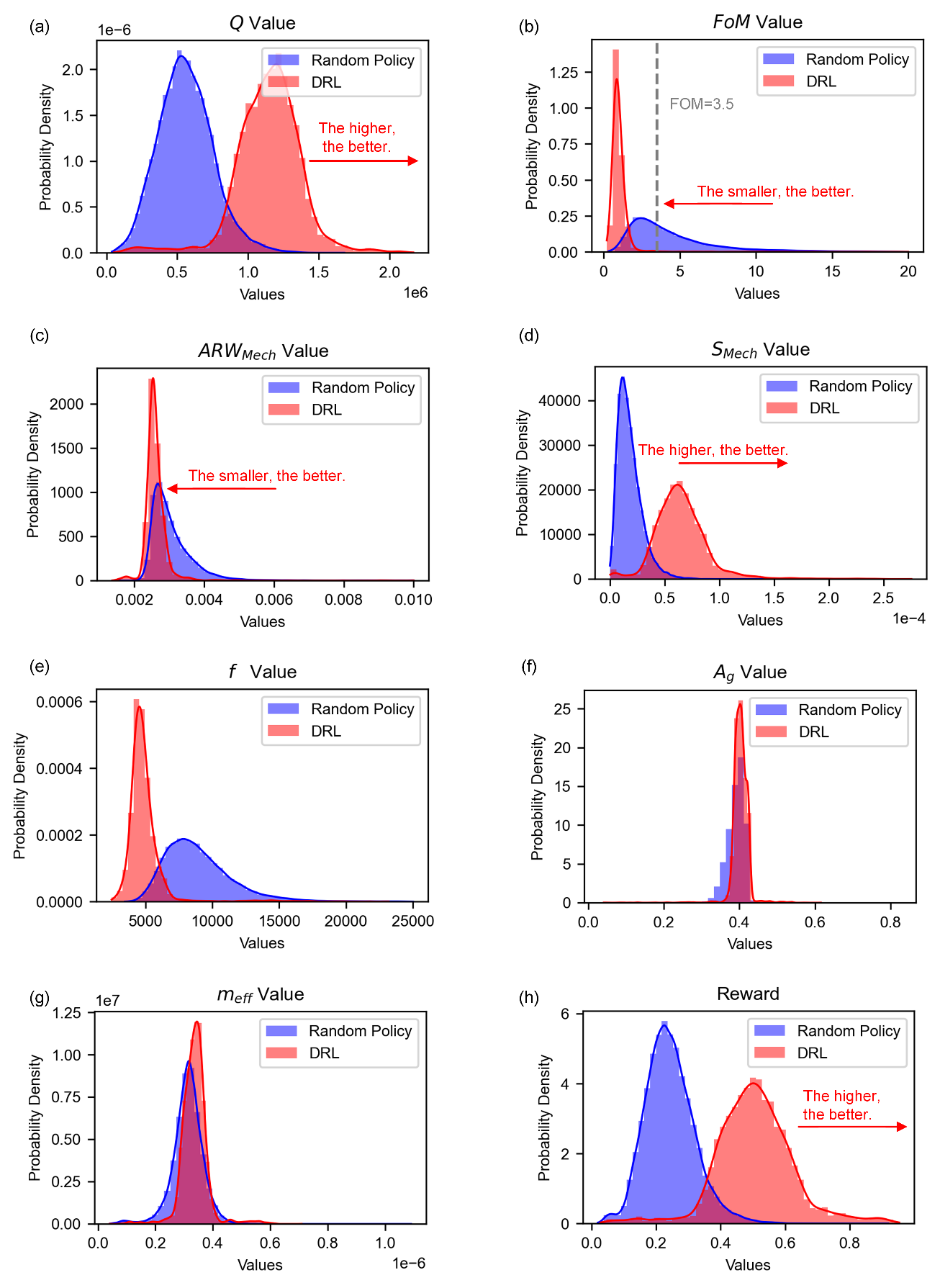


**Figure R1. Histogram of ground truth performance metrics.** (a) Quality factor. (b) Figure of merit. (c) Mechanical noise. (D) Mechanical sensitivity. (e) Frequency of the degenerate mode. (f) Angular gain coefficient. (g) Effective mass (h) Rewards.

## Supplementary Note S4: FoM specifications for navigation-grade MEMS DRGs

The robustness of the DRG to fabrication tolerance and disturbance can be measured via the *FoM*. The bias of the gyroscope can be approximated via Equation (R2).

$\Omega\approx FoM\times P$ (R2)

where P is a dimensionless parameter that measures the relative fabrication tolerance. The drift of bias can be approximated via Equation (R3).

$\partial\Omega\approx FoM\times\partial P$ (R3)

where *∂P* represents the instability of the relative fabrication tolerance within the operating temperature range, with typical values for silicon DRGs being approximately 0.8 ppm^32^. For the gyroscope to achieve navigation-grade bias stability (*∂Ω ≤ 0.01°/h*^32,46^), *FoM ≤ 3.5°/s* must be satisfied.

## Supplementary Note S5: Representative Structural Topologies


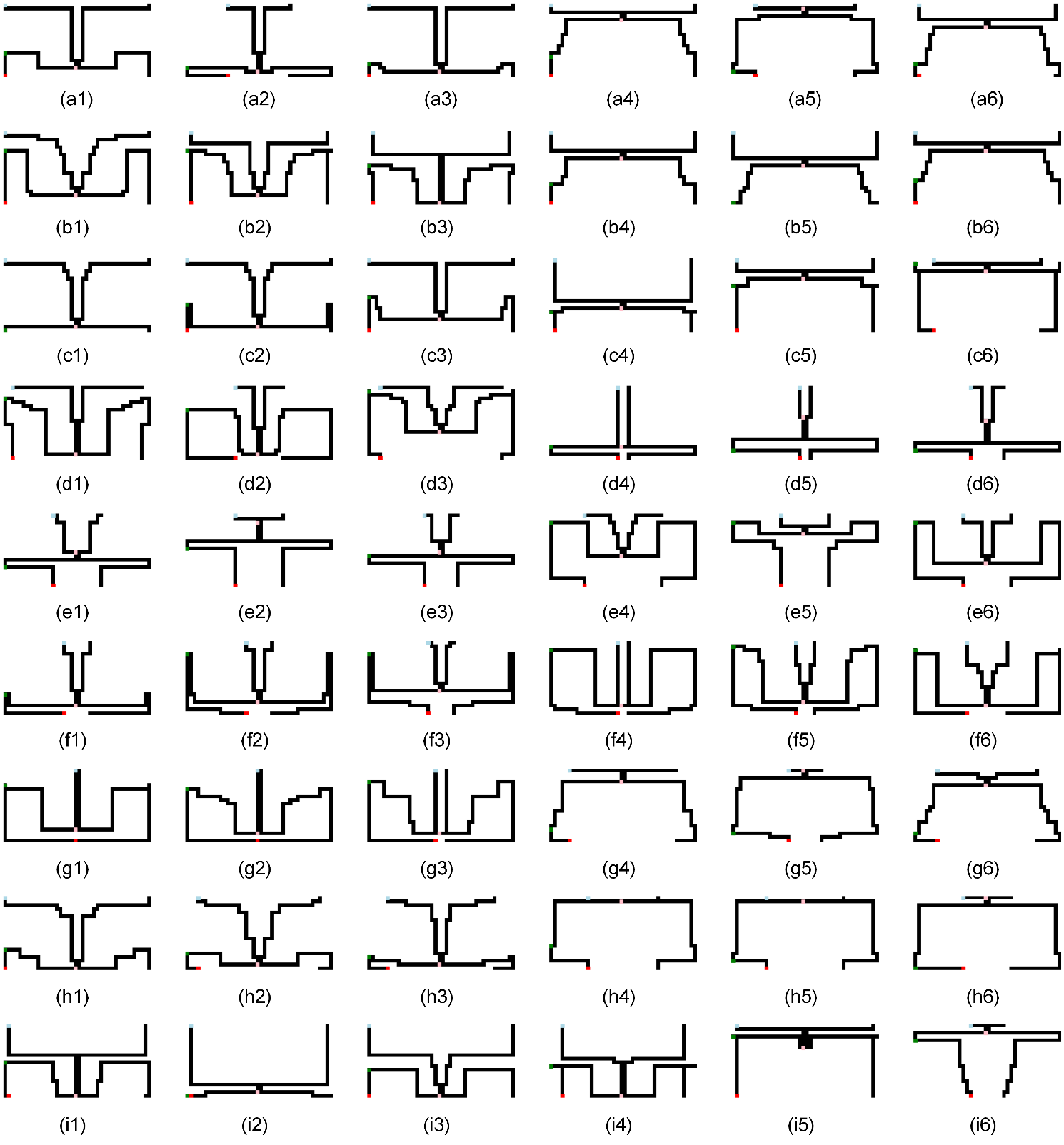


**Figure R2. Representative Topologies.** Black represents the topology, red represents the SP, green and pink represent the WP1 and the WP2, respectively, and light blue represents the EP.

We selected some representative topologies from the top-2000 rewards for demonstration. For observation, only the agents’ paths are displayed here. These paths can be mapped to topologies by employing the agent walking rules and mapping methods in the paper. Except for the i-th row, we deliberately grouped similar topologies together in sets of three.

The topologies designed by DRL are quite different from manual designs, demonstrating greater flexibility, irregularity, and a higher degree of freedom, which are hard to achieve by human experts. In these topologies, even though the endpoints are different, as in (a1), (a2), and (a3), the topologies finally exhibit similarities, which means that DRL is capable of learning the key features that affect the DRG performance, leading to analogous outcomes. Moreover, some topologies exhibit interesting patterns. For example, (d4)−(d6) look like a cross, (e1)−(e3) resemble a small person stretching out their arms, and (e4)−(f6) look like a person raising their arms, featuring heads and bodies of various shapes. These interesting similarities not only highlight the diversity of the discovered topologies but also reflect the complex dynamics behind them. The performance metrics of these topologies are listed in Table R2.

**Table R2.** Comparison of the mechanical performance metrics in Figure R2

| Topology | Q | *FoM* [*°/s*] | *S_mech_* [*m/(rad/s)*] | ARW_mech_ [°/$\sqrt{h}$] | *f* [Hz] | *m_eff_* [*kg*] | Reward |
| --- | --- | --- | --- | --- | --- | --- | --- |
| a(1) | 1.42×10^6^ | 0.61 | 9.42×10^-5^ | 0.0027 | 3885.1 | 2.88×10^-7^ | 0.646 |
| a(2) | 1.45×10^6^ | 0.62 | 9.31×10^-5^ | 0.0025 | 4011.2 | 3.22×10^-7^ | 0.656 |
| a(3) | 1.43×10^6^ | 0.59 | 9.63×10^-5^ | 0.0028 | 3789.8 | 2.88×10^-7^ | 0.653 |
| a(4) | 1.48×10^6^ | 0.59 | 9.74×10^-5^ | 0.0025 | 3977.5 | 3.12×10^-7^ | 0.676 |
| a(5) | 1.36×10^6^ | 0.73 | 7.87×10^-5^ | 0.0027 | 4288.5 | 2.91×10^-7^ | 0.578 |
| a(6) | 1.39×10^6^ | 0.73 | 7.89×10^-5^ | 0.0027 | 4338.4 | 2.92×10^-7^ | 0.584 |
| b(1) | 1.58×10^6^ | 0.51 | 1.12×10^-4^ | 0.0025 | 3664.3 | 3.33×10^-7^ | 0.749 |
| b(2) | 1.75×10^6^ | 0.43 | 1.34×10^-4^ | 0.0025 | 3503.7 | 2.94×10^-7^ | 0.851 |
| b(3) | 1.62×10^6^ | 0.50 | 1.14×10^-4^ | 0.0025 | 3713.5 | 3.08×10^-7^ | 0.759 |
| b(4) | 1.41×10^6^ | 0.63 | 9.07×10^-5^ | 0.0025 | 4154.0 | 3.05×10^-7^ | 0.643 |
| b(5) | 1.41×10^6^ | 0.67 | 8.56×10^-5^ | 0.0026 | 4176.5 | 3.00×10^-7^ | 0.616 |
| b(6) | 1.46×10^6^ | 0.59 | 9.65×10^-5^ | 0.0025 | 4041.0 | 3.10×10^-7^ | 0.673 |
| c(1) | 1.41×10^6^ | 0.63 | 9.04×10^-5^ | 0.0028 | 3955.3 | 2.77×10^-7^ | 0.627 |
| c(2) | 1.42×10^6^ | 0.62 | 9.20×10^-5^ | 0.0028 | 3922.3 | 2.86×10^-7^ | 0.636 |
| c(3) | 1.36×10^6^ | 0.67 | 8.49×10^-5^ | 0.0027 | 4116.4 | 2.93×10^-7^ | 0.605 |
| c(4) | 1.28×10^6^ | 0.84 | 6.82×10^-5^ | 0.0027 | 4646.2 | 3.04×10^-7^ | 0.532 |
| c(5) | 1.34×10^6^ | 0.73 | 7.90×10^-5^ | 0.0024 | 4498.5 | 3.12×10^-7^ | 0.592 |
| c(6) | 1.26×10^6^ | 0.81 | 7.09×10^-5^ | 0.0023 | 4786.7 | 3.29×10^-7^ | 0.557 |
| d(1) | 1.32×10^6^ | 0.55 | 1.05×10^-4^ | 0.0027 | 3351.6 | 3.49×10^-7^ | 0.679 |
| d(2) | 1.71×10^6^ | 0.41 | 1.41×10^-4^ | 0.0025 | 3190.4 | 3.41×10^-7^ | 0.872 |
| d(3) | 1.27×10^6^ | 0.64 | 8.90×10^-5^ | 0.0025 | 3769.8 | 3.69×10^-7^ | 0.619 |
| d(4) | 1.37×10^6^ | 0.72 | 7.92×10^-5^ | 0.0026 | 4336.3 | 3.12×10^-7^ | 0.588 |
| d(5) | 1.40×10^6^ | 0.65 | 8.75×10^-5^ | 0.0026 | 4178.6 | 2.93×10^-7^ | 0.624 |
| d(6) | 1.37×10^6^ | 0.66 | 8.71×10^-5^ | 0.0027 | 4098.5 | 2.94×10^-7^ | 0.617 |
| e(1) | 1.37×10^6^ | 0.70 | 8.18×10^-5^ | 0.0026 | 4231.5 | 3.14×10^-7^ | 0.598 |
| e(2) | 1.32×10^6^ | 0.74 | 7.78×10^-5^ | 0.0026 | 4368.0 | 3.13×10^-7^ | 0.579 |
| e(3) | 1.40×10^6^ | 0.64 | 8.99×10^-5^ | 0.0027 | 3991.2 | 2.86×10^-7^ | 0.627 |
| e(4) | 1.40×10^6^ | 0.60 | 9.59×10^-5^ | 0.0025 | 3875.3 | 3.32×10^-7^ | 0.662 |
| e(5) | 1.37×10^6^ | 0.69 | 8.31×10^-5^ | 0.0025 | 4295.4 | 3.12×10^-7^ | 0.607 |
| e(6) | 1.48×10^6^ | 0.58 | 9.93×10^-5^ | 0.0026 | 3895.0 | 2.99×10^-7^ | 0.679 |
| f(1) | 1.44×10^6^ | 0.62 | 9.27×10^-5^ | 0.0027 | 3978.6 | 2.86×10^-7^ | 0.644 |
| f(2) | 1.58×10^6^ | 0.54 | 1.06×10^-4^ | 0.0025 | 3724.7 | 3.54×10^-7^ | 0.724 |
| f(3) | 1.42×10^6^ | 0.65 | 8.83×10^-5^ | 0.0025 | 4017.9 | 3.60×10^-7^ | 0.635 |
| f(4) | 1.85×10^6^ | 0.37 | 1.53×10^-4^ | 0.0025 | 3105.6 | 3.39×10^-7^ | 0.937 |
| f(5) | 1.82×10^6^ | 0.39 | 1.49×10^-4^ | 0.0025 | 3144.1 | 3.43×10^-7^ | 0.917 |
| f(6) | 1.68×10^6^ | 0.45 | 1.27×10^-4^ | 0.0024 | 3467.3 | 3.32×10^-7^ | 0.819 |
| g(1) | 1.79×10^6^ | 0.04 | 1.43×10^-4^ | 0.0025 | 3263.1 | 3.16×10^-7^ | 0.887 |
| g(2) | 1.73×10^6^ | 0.43 | 1.32×10^-4^ | 0.0026 | 3300.6 | 3.29×10^-7^ | 0.836 |
| g(3) | 1.74×10^6^ | 0.44 | 1.29×10^-4^ | 0.0025 | 3458.8 | 3.23×10^-7^ | 0.831 |
| g(4) | 1.25×10^6^ | 0.77 | 7.42×10^-5^ | 0.0029 | 4184.9 | 2.89×10^-7^ | 0.542 |
| g(5) | 1.38×10^6^ | 0.69 | 8.29×10^-5^ | 0.0027 | 4188.3 | 2.93×10^-7^ | 0.598 |
| g(6) | 1.32×10^6^ | 0.80 | 7.16×10^-5^ | 0.0028 | 4550.5 | 2.83×10^-7^ | 0.546 |
| h(1) | 1.45×10^6^ | 0.62 | 9.26×10^-5^ | 0.0026 | 4055.5 | 2.92×10^-7^ | 0.649 |
| h(2) | 1.30×10^6^ | 0.81 | 7.04×10^-5^ | 0.0024 | 4647.0 | 3.59×10^-7^ | 0.557 |
| h(3) | 1.37×10^6^ | 0.71 | 8.10×10^-5^ | 0.0026 | 4316.8 | 2.92×10^-7^ | 0.593 |
| h(4) | 1.25×10^6^ | 0.84 | 6.81×10^-5^ | 0.0028 | 4629.3 | 2.67×10^-7^ | 0.521 |
| h(5) | 1.22×10^6^ | 0.86 | 6.69×10^-5^ | 0.0029 | 4604.4 | 2.66×10^-7^ | 0.511 |
| h(6) | 1.47×10^6^ | 0.58 | 9.80×10^-5^ | 0.0026 | 3825.5 | 3.13×10^-7^ | 0.672 |
| i(1) | 1.63×10^6^ | 0.49 | 1.16×10^-4^ | 0.0026 | 3673.9 | 2.92×10^-7^ | 0.762 |
| i(2) | 1.24×10^6^ | 0.82 | 6.96×10^-5^ | 0.0023 | 4839.7 | 3.34×10^-7^ | 0.551 |
| i(3) | 1.59×10^6^ | 0.51 | 1.12×10^-4^ | 0.0026 | 3734.6 | 2.85×10^-7^ | 0.740 |
| i(4) | 1.29×10^6^ | 0.80 | 7.20×10^-5^ | 0.0026 | 4692.6 | 2.74×10^-7^ | 0.550 |
| i(5) | 1.35×10^6^ | 0.69 | 8.32×10^-5^ | 0.0026 | 4164.0 | 3.10×10^-7^ | 0.601 |
| i(6) | 1.35×10^6^ | 0.72 | 7.99×10^-5^ | 0.0025 | 4257.8 | 3.41×10^-7^ | 0.593 |

## Supplementary Note S6: Performance Metrics in Figure 7

**Table R3.** Comparison of the mechanical performance metrics in Figure 6. FB_n_ is the topology with a flexible beam structure. DW_n_ is the topology with a double-wing structure. SS_n_ is the topology with a stepwise staircase structure.

| Topology | Q | *FoM* [*°/s*] | *S_mech_* [*m/(rad/s)*] | ARW_mech_ [°/$\sqrt{h}$] | *f* [Hz] | *m_eff_* [*kg*] | Reward |
| --- | --- | --- | --- | --- | --- | --- | --- |
| FB_1_ | 1.48×10^6^ | 0.57 | 9.97×10^-5^ | 0.0025 | 3885.7 | 2.88×10^-7^ | 0.69 |
| FB_2_ | 1.80×10^6^ | 0.40 | 1.44×10^-4^ | 0.0024 | 3262.1 | 3.22×10^-7^ | 0.90 |
| FB_3_ | 1.20×10^6^ | 0.91 | 6.27×10^-5^ | 0.0027 | 4889.3 | 2.88×10^-7^ | 0.50 |
| FB_4_ | 1.99×10^6^ | 0.32 | 1.81×10^-4^ | 0.0025 | 2833.0 | 3.12×10^-7^ | 1.06 |
| FB_5_ | 1.84×10^6^ | 0.37 | 1.56×10^-4^ | 0.0025 | 3141.4 | 2.91×10^-7^ | 0.95 |
| FB_6_ | 1.37×10^6^ | 0.69 | 8.35×10^-5^ | 0.0028 | 4176.3 | 2.92×10^-7^ | 0.60 |
| DW_1_ | 1.14×10^6^ | 0.83 | 6.89×10^-4^ | 0.0028 | 4122.7 | 3.33×10^-7^ | 0.51 |
| DW_2_ | 1.46×10^6^ | 0.64 | 9.00×10^-4^ | 0.0028 | 3999.8 | 2.94×10^-7^ | 0.63 |
| DW_3_ | 1.42×10^6^ | 0.66 | 8.65×10^-4^ | 0.0025 | 4228.7 | 3.08×10^-7^ | 0.63 |
| DW_4_ | 1.19×10^6^ | 0.87 | 6.59×10^-5^ | 0.0027 | 4635.4 | 3.05×10^-7^ | 0.51 |
| DW_5_ | 1.68×10^6^ | 0.46 | 1.25×10^-4^ | 0.0025 | 3529.1 | 3.00×10^-7^ | 0.81 |
| DW_6_ | 2.15×10^6^ | 0.29 | 1.98×10^-4^ | 0.0025 | 2809.3 | 3.10×10^-7^ | 1.14 |
| SS_1_ | 1.60×10^6^ | 0.52 | 1.10×10^-4^ | 0.0025 | 3712.4 | 2.77×10^-7^ | 0.74 |
| SS_2_ | 1.75×10^6^ | 0.43 | 1.34×10^-4^ | 0.0025 | 3509.6 | 2.86×10^-7^ | 0.85 |
| SS_3_ | 1.49×10^6^ | 0.58 | 9.91×10^-5^ | 0.0024 | 3817.3 | 2.93×10^-7^ | 0.69 |
| SS_4_ | 1.54×10^6^ | 0.56 | 1.03×10^-4^ | 0.0025 | 3762.7 | 3.04×10^-7^ | 0.71 |
| SS_5_ | 1.75×10^6^ | 0.34 | 1.67×10^-4^ | 0.0025 | 2798.2 | 3.12×10^-7^ | 0.98 |
| SS_6_ | 1.48×10^6^ | 0.60 | 9.54×10^-5^ | 0.0028 | 3896.9 | 3.29×10^-7^ | 0.66 |

## Supplementary Note S7: Architecture of the Neural Networks in this Work

For detailed information on these architectures, please refer to the corresponding section in the Methods section.


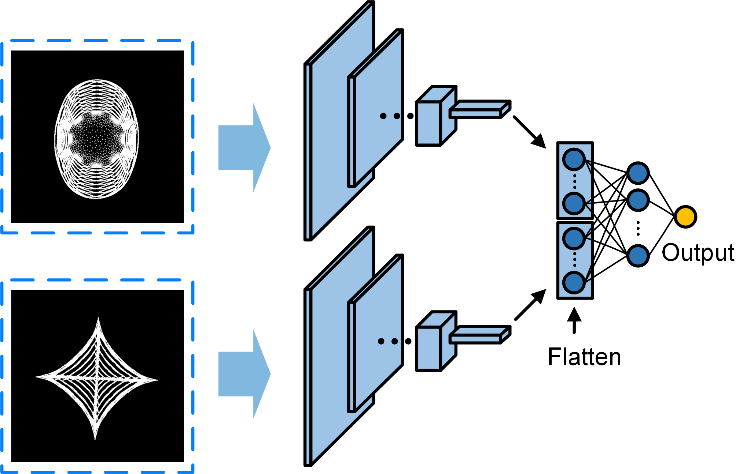


**Figure R3. Structure of the mode identification network.**


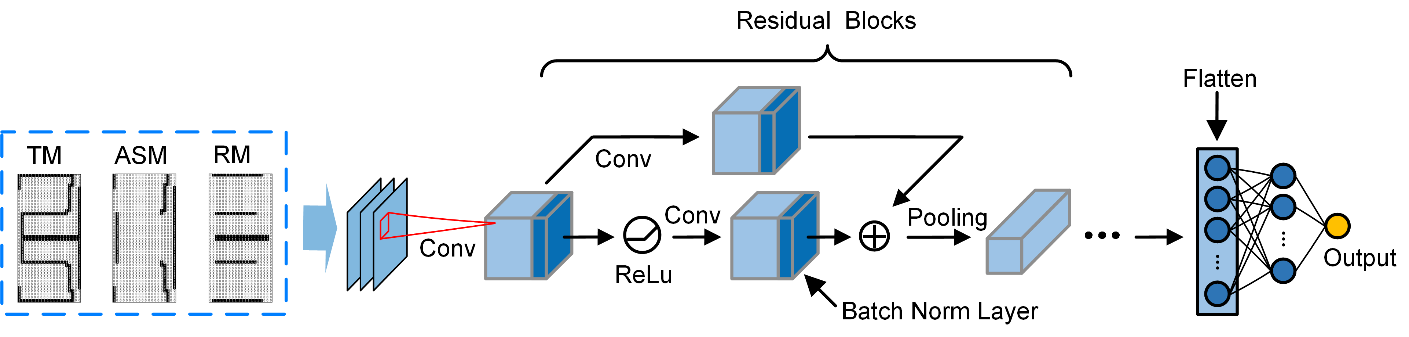


**Figure R4. Structure of the surrogate model.**


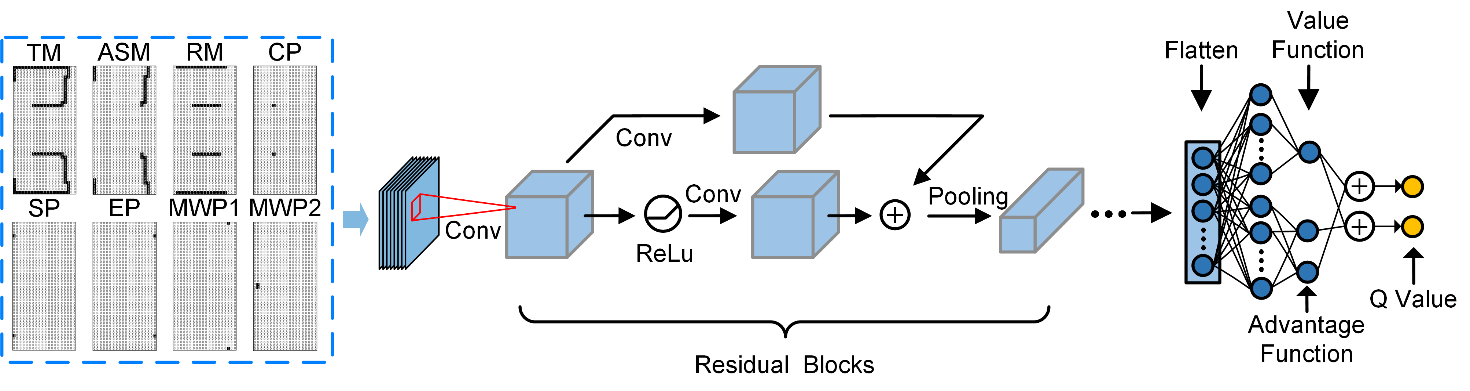


**Figure R5. Q-network structure.**

## Supplementary Note S8: Convergence curves of the mode identification neural network


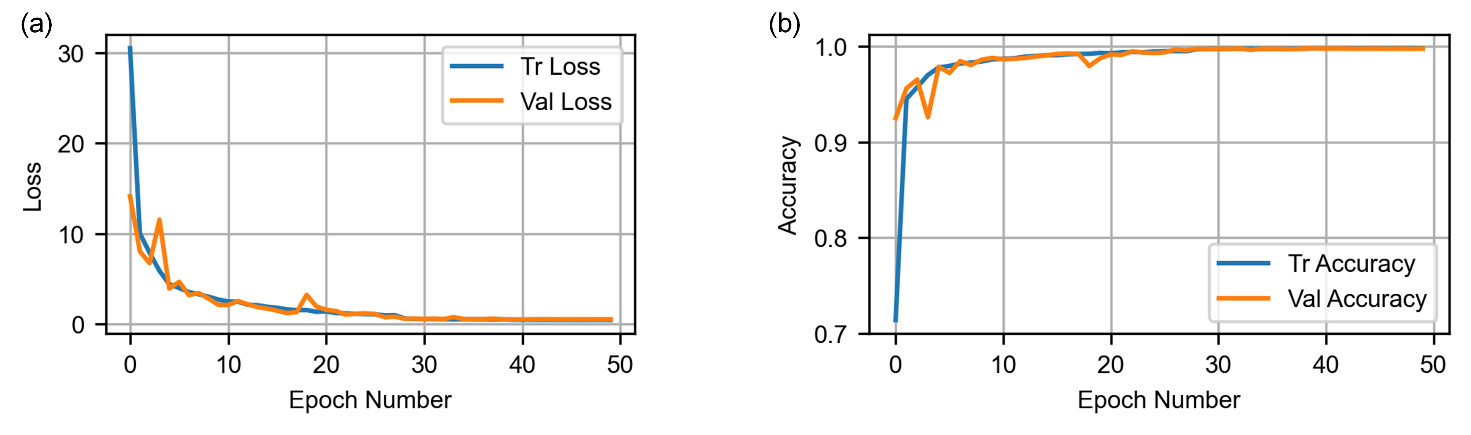


**Figure R6. Convergence curves of mode identification neural network training.** (a) Loss convergence curve. (b) Error rate convergence curve.

The convergence curve of the mode identification neural network is shown in Figure R6, demonstrating a stable convergence trend during training. In the loss curve (Figure R6(a)), both training and validation losses decrease rapidly in the initial phase and then steadily approach low values. For the accuracy curve (Figure R6(b)), both curves rapidly increase at the beginning of training, followed by slower growth and stabilization, fluctuating at a high level. The final training loss is 0.45, and the validation loss is 0.53. The final training accuracy is 99.80%, and the final validation accuracy is 99.77%. This finding indicates that the neural network achieves consistently high performance on both training and validation data without showing signs of overfitting or underfitting.

## Supplementary Note S9: Convergence Information of Surrogate Models

Table R4 presents the final training results of the surrogate models. Given the long-tail distribution of the data, training the surrogate model to directly predict *S_mech_*, *FoM*, and *ARW_mech_* was less accurate than using formulas for calculations. Figure R7 displays the convergence curves of various models, with the left side showing loss, the middle showing error, and the right side showing the R2 score. The solid lines represent the validation set, whereas the dashed lines represent the training set. A dynamic learning rate approach was adopted, and while fluctuations occurred in the early stages of training, stable convergence was ultimately achieved.

**Table R4.** Training results of surrogate models. The "(compute)" notation indicates that the results are calculated according to Equations (4)‑(6).

|  | Training Set | | | Validation Set | | |
| --- | --- | --- | --- | --- | --- | --- |
|  | Loss | Error | R2 | Loss | Error | R2 |
| *Q* | 0.0125 | 0.0039 | 0.9964 | 0.0966 | 0.0242 | 0.9931 |
| *ARW_mech_* | 0.0345 | 0.0065 | 0.9805 | 0.1777 | 0.0294 | 0.9757 |
| *ARW_mech_ (compute)* | - | - | - | - | 0.0204 | 0.9757 |
| *S_mech_* | 0.0361 | 0.0092 | 0.9966 | 0.2644 | 0.0522 | 0.9936 |
| *S_mech_ (compute)* | - | - | - | - | 0.0342 | 0.9941 |
| *FoM* | 0.5424 | 0.0351 | 0.9790 | 3.4563 | 0.1495 | 0.9726 |
| *FoM (compute)* | - | - | - | - | 0.0342 | 0.9823 |
| *f* | 0.0087 | 0.0035 | 0.9967 | 0.0553 | 0.0166 | 0.9948 |
| *A_g_* | 0.0041 | 0.0022 | 0.9693 | 0.0184 | 0.0096 | 0.9660 |
| *m_eff_* | 0.0116 | 0.0036 | 0.9866 | 0.0654 | 0.0184 | 0.9813 |


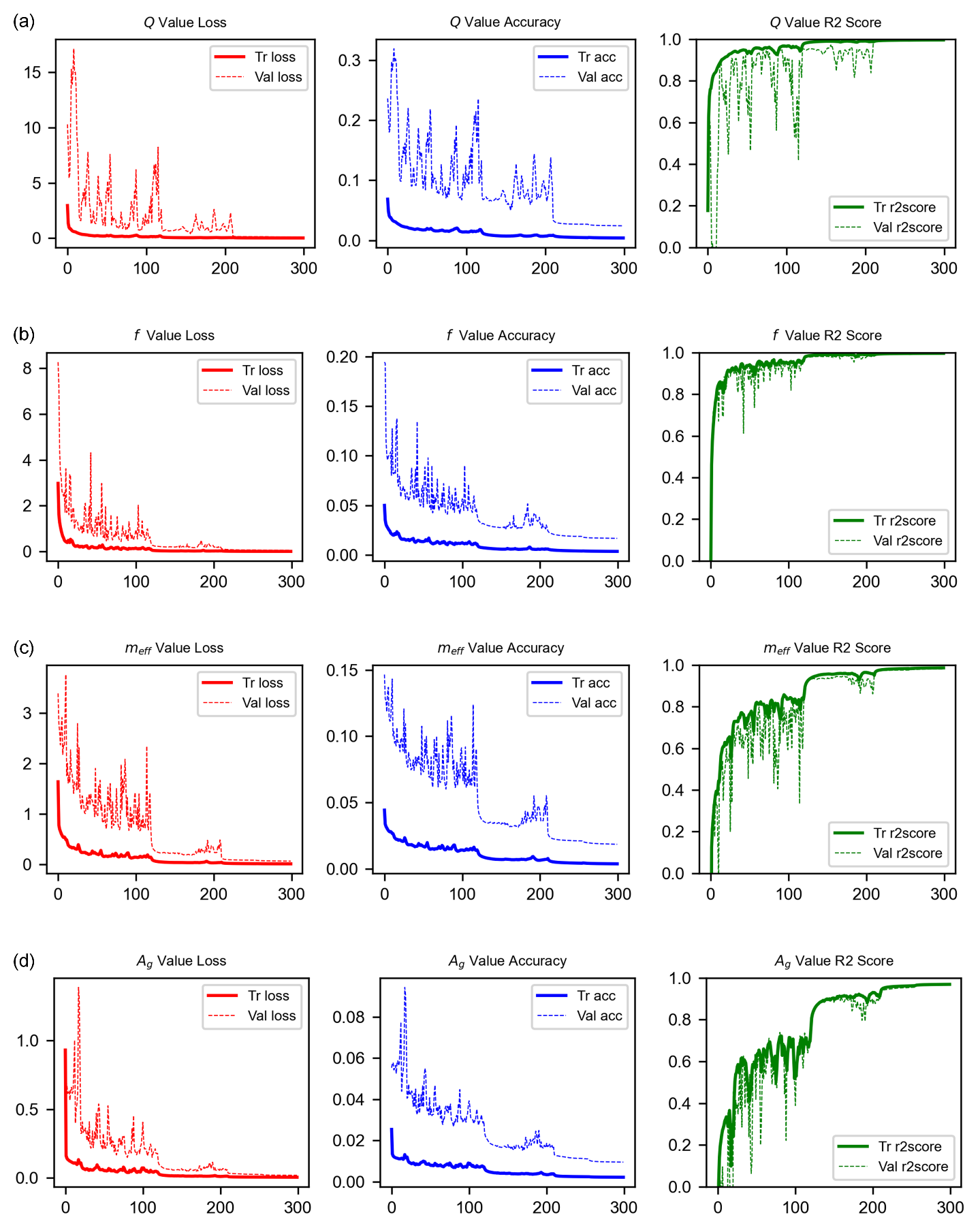


**Figure R7. Training loss and validation loss during the 300-epoch training process.** **(a)** *Q* value. **(b)** *f* value for calculating *S_mech_*. **(c)** *m_eff_* value for calculating *ARW_mech_*. **(d)** Ag values for calculating *S_mech_* and *A*
